# Prevalence and associated risk factors of preterm birth among neonates in referral hospitals of Amhara Region, Ethiopia

**DOI:** 10.1371/journal.pone.0276793

**Published:** 2022-10-27

**Authors:** Dagnew Getnet Adugna

**Affiliations:** Department of Human Anatomy, School of Medicine, College of Medicine and Health Science, University of Gondar, Gondar, Ethiopia; Medical Research Council, SOUTH AFRICA

## Abstract

**Introduction:**

Preterm birth (PTB) is the largest direct cause of neonatal mortality and the second leading cause of under-five mortality following pneumonia. Although there are studies conducted before, the magnitude of PTB remains a major issue in most developing countries including Ethiopia. Therefore, this study aims to assess the prevalence and associated factors of premature birth among newborns delivered in Amhara Region Referral Hospitals, Northern Ethiopia.

**Methods:**

A hospital-based cross-sectional study was undertaken from February to April 2020. A systematic sampling technique was used to select 482 mother-newborn pairs. The data were collected by interviewing the mothers and reviewing their charts using a structured and pretested questionnaire. The outcome variable was preterm birth. Data were entered using Epi-data version 4.6 and then analyzed using STATA software (version 14). Bivariable and multivariable logistic regression analyses were done to determine the risk factors associated with premature birth.

**Results:**

In this study, the prevalence of premature birth was 11.41% (95% CI: 8.9, 14.6%). In multivariable logistic regression model; maternal age < 20 years (Adjusted odds ratio (AOR) = 7.8: 95% CI 2.3–26), preeclampsia (AOR = 5: 95% CI 2.3–11), premature rupture of membrane (AOR = 3.9: 95%CI 1.6–9.0), chronic medical illness (AOR = 4.6:95% CI2.1–10), and history of stillbirth (AOR = 2.7: 95% CI 1.1–7.3) were significantly associated with preterm birth.

**Conclusion:**

The study indicates preterm birth is a major public health problem among newborns delivered in Amhara region referral hospitals. The risk factors associated with preterm birth are maternal age <20 years, preeclampsia, premature rupture of membranes, chronic medical illness, and history of stillbirth. Therefore, public health interventions have to be made to reduce the burden of prematurity through early detection and management of preeclampsia, premature rupture of membranes, and chronic medical illness. Obstetric care providers should give due attention to women with an age of <20 years and a history of stillbirth.

## Introduction

Preterm birth (PTB) is defined as a live birth that occurred before 37 complete weeks of gestation or less than 259 days from the first day of the last normal menstrual period (LNMP) [1, 2]. It is the largest direct cause of neonatal mortality and the second leading cause of under-five mortality following pneumonia [3].

Worldwide, about 15 million (more than one in 10) babies are born preterm each year and about 12 million (81.1%) of this prematurity occurs in Asia and sub-Saharan Africa [4]. More than one million neonates die due to prematurity every year [2]. PTB affects 10.6% of all newborns in North America followed by 6.2% in Europe but in Africa, the prevalence is highest (11.9%) [5]. The estimated prevalence of PTB in developing countries (12%) is higher compared with developed countries (9%) [5, 6]. Generally, the prevalence of PTB varies from country to country among different studies. For example, the prevalence of PTB in Iran is 5.1% [7], and in Sweden 5% [8]. The prevalence of PTB in Nigeria is 12% [9], in Algeria 9.6% [10] and Kenya 18.3% [11]. In Ethiopia, the magnitude of PTB varies from 4.4% to 25.9% [12–17].

Previous studies conducted in different regions indicated that several risk factors were identified for preterm birth. This includes preeclampsia, premature rupture of membrane (PROM), antepartum hemorrhage (APH)), low income, large family number (≥4), educational status, and rural area residence [7, 9, 11, 13, 16–21]. Other factors that increase the risk of preterm birth incude maternal age <20 years, history of stillbirth, history of abortion, history of preterm, lack of antenatal care visit (ANC), short birth space, human immunodeficiency virus/ acquired immune deficiency syndrome (HIV/AIDS), anemia, visible congenital anomalies, induced labor, and chronic illness [16, 19, 22–24]. Multiple pregnancies [25], maternal cardiovascular disease [26], and polyhydraminious [27] were also predisposing factors of PTB.

PTB babies are affected by long-term or short-term complications such as difficulty of breathing, feeding problems, cerebral palsy, the effect on brain development later in life, visual and hearing impairment, and poor prognosis. PTB has significant economic impacts at the individual, family, and social levels [5, 28]. The survival rate of preterm babies in developed countries is higher compared to developing countries. For instance, over 90% of extremely preterm babies (< 28 weeks) born in developing countries die within the first few days of life; but < 10% of extremely preterm babies die in developed countries [29]. The difference may be due to better neonatal care setup and low psychosocial inequality in higher-income countries than in developing nations [30, 31].

PTB is an important public health problem in Ethiopia. However, in most low-income countries, including Ethiopia, little emphasis is given to PTB intervention as a means of reducing infant mortality. Although there are few studies conducted before in some regions of Ethiopia, the magnitude and possible risk factors of PTB are varied from region to region. In addition, our study addresses some methodological issues (appropriate sample size calculation, sampling technique, and multicenter study area) that the previous studies didn’t consider. Conducting the study at a large regional level is very important to designing effective public health programs and interventions. Therefore, this study aims to assess the prevalence and associated factors of PTB among newborns delivered in Amhara Region Referral Hospitals, Northern Ethiopia.

## Materials and methods

### Study design, period, and study setting

An institution-based cross-sectional study was conducted in three randomly selected Referral Hospitals in the Amhara Region between February and April 2020. The region has 67 public hospitals, 734 health centers, and 2941 health posts [32]. There are seven referral hospitals, which serve more than 22 million people in the Amhara region, Ethiopia. These include the University of Gondar Comprehensive Specialized Hospital, Felegehiwot Comprehensive Specialized Hospital, Dessie Referral Hospital, Debre-Markos Referral Hospital, Debre-Tabor referral Hospital, Woldia Referral Hospital, and Debre-Birhan Referral Hospital. Each referral hospital provides services for more than five million people, contains 200 to 400 beds, and reports 2000 to 4000 deliveries each year and five to ten deliveries per day. Of these, the University of Gondar Comprehensive Specialized Hospital, Debre Tabor Referral Hospital, and Bahir Dar Felege-Hiwot Comprehensive Specialized Referral Hospitals were selected randomly using a lottery method.

### Populations

The source population was all newborn-mother pairs delivered in the referral hospitals of the Amhara Region and the study population was all newborn-mother pairs delivered in the selected referral hospital during the study period.

### Inclusive and exclusive criteria

All newborn-mother pairs delivered at the selected public referral hospitals during the study period were included. Those mothers with unknown LNMP or absent early pregnancy ultrasound evidence (≤20 completed weeks of gestation) for gestational age (GA) calculation were excluded.

### Sample size determination and sampling procedure

The sample size was calculated using a single population proportion formula by considering the confidence level (95%), the margin of error = 3%, and 11.6% prevalence taken from the previous study done in Debremarkose, Ethiopia [33].


$$n=\frac{{\left(Z_{\alpha /2}\right)}^{2}*p(1-p)}{d^{2}}\;\text{n}=\frac{{\left(1.96\right)}^{2}*(0.116)*(1-0.116))}{{\left(0.03\right)}^{2}}=438$$

By adding a 10% nonresponse, the final sample size was 482. From the seven referral hospitals providing labor and delivery service in the Amhara Region, we selected three referral hospitals randomly. The sample size was proportionally allocated to each selected referral hospital before the beginning of actual data collection time, based on the previous hospital delivery report. The study participants were selected from each hospital using a systematic sampling technique.

### Study variables

#### Dependent variable

Preterm birth.

#### Independent variables

Socio-demographic, obstetric, medical, and newborn-related factors have been included. These are maternal age, marital status, educational status, residence, family size, occupational status, average monthly income, preeclampsia, APH, PROM, history of preterm birth, history of abortion, history of stillbirth, history of cesarean section, parity, interpregnancy interval, ANC follow-up status, the number of ANC visits, the onset of labor, chronic medical illness (maternal HIV, anemia, cardiac disease, and chronic kidney disease), and the sex of newborns.

### Operational definition

#### Preterm birth

A birth before 37 completed weeks of gestation but after 28 weeks (fetal viability).

#### Family size

Number of family members such as her husband and number of children living together in one home.

### Data collection procedure and tools

The data were collected using a structured questionnaire through face-to-face interviews and reviewing the charts of the mothers. Socio-demographic and obstetric variables were collected by interviewing the mothers during the postpartum period. The client’s medical records were reviewed to obtain medical-related variables. The questionnaire was adopted from different literature. It was first developed in English then translated into Amharic and translated back into English. The last normal menstrual period (LNMP) was obtained from the mother’s medical records. If it was not documented in the chart, the mothers were interviewed about their LNMP during the postpartum period. GA has been calculated based on her LNMP date or early pregnancy ultrasound report (up to and including 20 completed weeks of gestation). Newborns delivered before 37 completed weeks of gestation but after viability (28 weeks of gestation) were categorized as preterm.

### Data quality control

Before the actual data collection period, training was provided for data collectors. The questionnaires were pretested to 5% of the sample size at Debark General Hospital. The data was collected by six B.sc midwives. Data collectors were supervised daily by supervisors, and the collected data were checked for completeness, consistency, and clarity before entry.

### Data processing and analysis

The data were checked, coded, and entered into Epidata version 4.6, and exported to STATA version 14 software for analysis. Descriptive statistics like percentages, proportions, and mean are used. The results were presented in tables and text. A Chi-square assumption was done for each categorical independent variable. Analysis was conducted using binary logistic regression to determine the risk factors associated with PTB. Both bivariable and multivariable binary logistic regression analyses were employed. Model fitness was assessed using the Hosmer-Lemeshow test. Variables with a p-value of less than 0.2 in the bivariable logistic regression were considered for the multivariable logistic regression analysis. In the multivariable logistic regression analysis, the Adjusted Odds Ratio (AOR) with a 95% confidence interval was calculated. Lastly, variables with a p-value of less than 0.05 were considered significant.

### Ethical considerations

Ethical clearance was obtained from the ethical review committee of the School of Medicine, College of Medicine and Health Sciences, University of Gondar. An official letter was submitted to the University of Gondar Comprehensive Specialized Hospital, Debre Tabor Hospital, and Bahirdar Felege Hiwot Comprehensive Specialized Hospital. Written informed consent was taken from the study participants after a clear explanation of the purpose of the study. Confidentiality was maintained. This study was carried out in accordance with the Declaration of Helsinki. University of Gondar Ethical Review Committee approved participants under the age of 18 years to provide informed consent on their behalf, and the informed consent included the publication of anonymized responses.

## Results

### Socio-demographic characteristics of the study participants

A total of 482 study participants were enrolled in the study with a response rate of 100%. The mean age (±SD) of the mothers was 28.25 (±5.3) years. About 63.7% of participants were between the age group of 20–30 years. Most 365 (75.7%) participants were urban residents. In addition, 452(93.78%) of the mothers were married, 282 (58.5%) had secondary education, and 233 (48.5%) were housewives. About 76.35% of the study participants had less than five family numbers (Table 1).

**Table 1:** Socio-demographic characteristics of the study participants in governmental referral hospital, Amhara Region, Northwest Ethiopia 2020 (n = 482).

Variables	Category	Frequency	Percent (%)
Age of the mother (years)	<20	19	3.9
	20–30	304	63.7
	≥31	156	32.4
Residence of the participants	Urban	365	75.7
	Rural	117	24.3
Ethnicity	Amhara	467	97
	Tigrie	2	0.4
	Kimant	13	2.6
Religion	Orthodox	438	90.9
	Muslim	44	9.1
Marital status	Married	452	93.8
Educational status	Single	17	3.5
	Divorced and separated	13	2.7
	No formal education	135	28
	Primary Education (1–8)	65	13.5
	Secondary education and above	282	58.5
Mothers occupational status	Housewife	233	48.4
	Government employee	121	25.2
	Nongovernment employee	8	1.7
	Self-employed business	89	18.6
	Daily laborer	12	2.5
	Students and unemployed	18	3.6
Monthly income in Ethiopian Birr	≤1210	12	2.5
	1211–8970	378	78.4
	>8970	92	19.1
Number of Family members	≤4 members	368	76.3
	>4 members	114	23.7

### Maternal obstetric, medical, and newborn characteristics

In our study, the majority of 451 (93.4%) mothers had ANC follow-up during the current pregnancy. Of these, 363 (78.1%) had at least four visits. About 175 (36.3%) mothers were primiparous. Among the total mothers who gave birth during the study period, 48 (10%) had PROM, 55 (11.4%) had preeclampsia, and 40 (8.3%) had APH. Concerning the mode of delivery, 264 (54.8%) mothers were delivered by spontaneous vaginal delivery. Moreover, 48 (10%) had a previous history of stillbirth. Out of the total neonates, about 470 (97.5%) were live births (Table 2).

**Table 2:** Maternal obstetric, medical and newborn characteristics of participants in governmental referral hospital, Amhara Region, Northwest, Ethiopia 2020 (n = 482).

Variables	Category	Frequency	Percent (%)
ANC follow up	Yes	451	93.4
	No	31	6.6
Number of ANC visit	< 4 times	99	21.9
	≥ 4 times	352	78.1
Pregnancy status	Wanted and planned	347	72
	Wanted but unplanned	114	23.6
	Unwanted and unplanned	21	4.4
Dietary counseling during pregnancy	Yes	425	88.2
	No	57	11.8
Parity	Primiparous	175	36.3
	Multiparous	307	63.7
GA at delivery	Preterm	55	11.4
	Term	417	86.5
	Post-term	10	2.1
PROM	Yes	48	10
	No	434	90
Preeclampsia	Yes	55	11.4
	No	427	88.6
APH	Yes	40	8.3
	No	442	91.7
Chronic medical illness	Yes	65	13.5
	No	417	86.5
Types of medical illness	HIV	13	20
	Anemia	26	40
	Urinary tract infection	10	15.4
	Cardiac disease	3	4.6
	Others*	13	20
Previous history of PTB	Yes	36	7.5
	No	446	92.5
Previous history of stillbirth	Yes	48	10
	No	434	90
Previous history of abortion	Yes	44	9.1
	No	438	90.9
Modes of delivery	Spontaneous vaginal delivery	264	54.8
	Cesarean section	203	42.1
	Instrumental delivery	15	3.1
Birth weight (g) of newborn	<2500	64	13.3
	≥2500	418	86.7
Sex newborn	Male	272	56.4
	Female	210	43.6
Neonatal death	Yes	12	2.5
	No	470	97.5

Others* = renal disease and malaria

### Prevalence of PTB

In the present study, the prevalence of PTB was 11.4% (95% CI: 8.9, 14.6%) (Table 2).

### Factors associated with PTB

Binary logistic regression analysis was done using odds ratios (OR) and 95% CI. In the bivariable analysis; maternal age, residence, family size, ANC visits, preeclampsia, PROM, APH, chronic illness, previous history of stillbirth, previous history of abortion, and previous history of PTB were significantly associated with PTB at a p-value of 0.2. However, in the multivariable logistic regression analysis; maternal age <20 years, preeclampsia, PROM, chronic medical illness during pregnancy (HIV, anemia, chronic kidney, and cardiac disease), and history of stillbirth were found to be significantly associated with PTB.

The likelihood of PTB among mothers in the age group of <20 years was eight times higher compared to the mother’s age group of 20–30 years (AOR = 7.8:95% CI 2.3–26). Mothers with preeclampsia during pregnancy were about 5 times higher to give PTB than those who had no preeclampsia (AOR = 5:95% CI 2.3–11). The odds of a mother with PROM were about 4 times higher chance to give preterm babies than mothers with no PROM (AOR = 3.9:95% CI 1.6–9.0).

Mothers who were exposed to chronic medical illness during pregnancy had 5-fold higher odds of PTB compared to those who were not exposed to any medical illness during this pregnancy (AOR = 4.6: 95% CI 2.1–10). Similarly, mothers with a history of stillbirth before this indexed pregnancy had 3 times higher odds of PTB compared to those mothers who did not have a history of stillbirth (AOR = 2.7:95% CI 1.1–7.3) (Table 3).

**Table 3:** Factors associated with PTB among (n = 482) newborns delivered in governmental referral hospital, Amhara Region, Northwest Ethiopia 2020.

Variables	Category	Preterm birth		COR (95%CI)	AOR (95%CI)
		YES	No		
Age (years)	<20	7 (12.7%)	12 (2.8%)	5.3(2–14)*	7.8(2.3–26)*
	20–30	30(54.6%)	277 (64.9%)	1	1
	≥31	18 (32.7%)	138 (32.3%)	1.2 (0.6–2.2)	0.4(0.46–1.0)
Residence	Urban	31(56.4%)	334 (78.2%)	1	1
	Rural	24 (43.6%)	93 (21.8%)	2.8(1.5–4.9)*	1.5(0.7–3.2.0)
Family number	≤ 4	35(63.6%)	333(78%)	1	1
	>4	20(36.4%)	94 (22%)	2(1.1–3.6)*	1.9(0.8–4.9)
ANC visit	Yes	44 (80%)	406 (95.1%)	1	1
	No	11(20%)	21 (4.9%)	4.8 (2.1–10.6)*	2(0.7–6.0)
Preeclampsia	Yes	19(34.6%)	36(8.4%)	5.7 (2.9–11)**	5(2.3–11) **
	No	36 (65.4%)	391 (91.6%)	1	1
PROM	Yes	18(32.7%)	30 (7%)	6.4(3.2–12)**	3.9(1.6–9.0)*
	No	37(67.3%)	397(93%)	1	1
APH	Yes	9(16.4%)	31(7.3%)	2.4(1.1–5.6)*	1.7(0.6–4.6)
	No	46(83.6%)	396 (92.7%)	1	1
Chronic medical illness in pregnancy	Yes	18(32.7%)	47(11%)	3.9 (2–7.4)**	4.6(2.1–10)**
	No	37(67.3%)	380 (89%)	1	1
Previous history of stillbirth	Yes	10(18.2%)	38 (8.9%)	2.2(1.0–4.8)*	2.7(1.1–7.3)*
	No	45(81.8%)	389 (91.1%)	1	1
Previous history of PTB	Yes	8 (14.6%)	28 (6.6%)	2.4(1–5.6)*	1.8(0.6–5.6)
	No	47 (85.4%)	399 (93.4%)	1	1
Previous history of abortion	yes	12 (21.8%)	32 (7.5%)	3.4(1.6–7.1)*	1.7(0.7–4.6)
	No	43 (78.2%)	395 (92.5%)	1	1

1 = reference category,

* Statistically significant at p<0.05,

** p-value <0.001

## Discussion

This study aimed to assess the prevalence of PTB and its associated risk factors among newborns delivered in Referral Hospitals in the Amhara region. In this study, the overall prevalence of PTB was 11.41%. This is in line with studies conducted in Africa (11.9%) [5], North America (10.6%) [5], Tanzania (14.2%) [34], and Nigeria 12% [9]. A similar finding was also obtained from studies at Axum, Tigray region (13.3%) [23], and Debretabor town, Ethiopia (12.8%) [15]. This similarity between the present study and the previous studies in Axum and Debretabor may be due to various related levels of socioeconomic status and lifestyle of the respondents since all are from low-income and middle-income countries.

The result of this study is lower than the reports conducted in Kenya (18.3%) [11] and Jimma, Ethiopia (25.9%) [13]. The reason for this variation might be due to the difference in the health-seeking behavior of the study participants and methodological differences. However, the finding in this study is significantly higher than other studies conducted elsewhere [7, 8, 12, 35]. The possible explanation for this variation could be due to the difference in the study time, inclusion and exclusion criteria, quality of health services, and socio-demographic characteristics.

The odds of giving PTB were higher among mothers who had preeclampsia, maternal age less than 20 years, PROM, chronic medical illness during pregnancy, and history of stillbirth.

Our findings revealed that the likelihood of PTB among mothers in the age group of less than 20 years was eight times higher compared to the mother’s age group of 20–30 years. This is consistent with a systematic review and meta-analysis conducted in East Africa [36]. The study is also supported by studies done in Canada [37] and Ethiopia [22]. This might be due to as the age of mothers increases, their health-seeking behavior, and knowledge about pregnancy-related health problems will also be raised. Moreover, young women are more prone to many risk behaviors like alcohol consumption and less adherence to advice and counseling given by their health professionals compared to elder women [22].

Our study revealed that mothers who had preeclampsia had a 5 times increased risk of PTB than those who had no preeclampsia. The finding of this study is similar to the other studies carried out in Southern India [38], Kenya [11], Nigeria [9], and Ethiopia [18, 19, 33, 39]. This might be due to the complications of hypertension disease that can cause vascular damage to the placenta or decrease the uteroplacental blood flow [40]. This induces oxytocin receptors and results in intrauterine growth restriction that causes preterm labor and delivery.

This study indicated that mothers who had PROM had a four times higher chance to give preterm birth than those with no PROM. The finding of this study is consistent with the study done in Ghana [20], Nigeria [9], Kenya [11], and Ethiopia [15, 19]. This is justified by PROM may increase the fetal plasma interleukin-6 that will activate the spontaneous preterm labor [41]. Furthermore, this might be explained by the influence of the membrane rupturing on uterine contraction. The research evidence claims that some endogenous uterotonic hormones are released when the membrane ruptures and these hormones, in turn, induce uterine contractions triggering PTB.

Moreover, this study revealed that mothers who were exposed to chronic medical illnesses (HIV, anemia, chronic kidney disease, and cardiac disease) during pregnancy had 5-fold higher odds of PTB compared to those who were not exposed to any medical illness. This finding is supported by previous studies conducted in Ethiopia [13, 33, 39], and a systematic review and meta-analysis of East Africa [36]. This might be due to medical disorders during or before pregnancy affecting the placenta and the membrane which reduces the placental flow of oxygen and nutrients to the developing fetus in utero and thus increases the risk of preterm birth [42].

In this study, mothers who had a history of stillbirth had 3 times higher odds of PTB compared to those mothers who did not have a history of stillbirth. This study is in agreement with other studies done in Sidama, Southeast Ethiopia [19], and a study conducted in Jimma, Southwest Ethiopia [13]. This might be due to the recurrence of stillbirth in some women who initiate preterm labor in the preceding pregnancy.

### Limitations of the study

Being a cross-sectional study does not confirm a definitive cause-and-effect relationship. Since the study was hospital-based, it may not clearly show the real picture of PTB in the area.

## Conclusion

PTB is a major public health problem among newborns delivered in Amhara region referral hospitals. The risk factors associated with PTB are maternal age <20 years, preeclampsia, PROM, chronic medical illness during pregnancy, and history of stillbirth. Therefore, public health interventions have to be made to reduce the magnitude of PTB through early detection and management of preeclampsia, PROM, and chronic medical illness. Obstetric care providers should give due attention to women with an age of < 20 years and a history of stillbirth.

## Abbreviations

ANC: Antenatal Care
AOR: Adjusted Odds Ratio
APH: Antepartum hemorrhage
CI: Confidence Interval
COR: Crude Odds Ratio
GA: Gestational Age
HIV/AIDS: Acquired Immune Deficiency Syndrome/Human Immune Deficiency Virus
LNMP: Last Normal Menstrual Period
PROM: Premature Rupture of Membrane
PTB: Preterm Birth
STATA: Statistics/Data analysis
